# Supplementary material for: Occupational physical activity as a determinant of daytime activity patterns and pregnancy and infant health
Source: PLoS One. 2023 Dec 22;18(12):e0296285. doi: 10.1371/journal.pone.0296285 (PMC10745165; doi:10.1371/journal.pone.0296285)
Supplement: S1 Table — Data are presented as mean (sd). (DOCX) [file pone.0296285.s001.docx]

|  | **Average Wear Days** | **Average Wear Hours per Day** |
| --- | --- | --- |
| **activPAL** | | |
| Trimester 1 (n=122) | 6.9 (0.9) | 14.9 (1.0) |
| Trimester 2 (n=117) | 6.9 (1.1) | 15.1 (1.1) |
| Trimester 3 (n=111) | 6.9 (1.0) | 15.0 (1.0) |
| **ActiGraph** | | |
| Trimester 1 (n=122) | 7.5 (1.4) | 14.6 (1.2) |
| Trimester 2 (n=117) | 7.2 (1.1) | 14.5 (1.2) |
| Trimester 3 (n=107) | 7.2 (0.9) | 14.5 (1.5) |

**Supplemental Table 1. Average Wear Times for the activPAL and ActiGraph Monitors by Trimester**

Data are presented as mean (sd)
