# Supplementary material for: Occupational physical activity as a determinant of daytime activity patterns and pregnancy and infant health
Source: PLoS One. 2023 Dec 22;18(12):e0296285. doi: 10.1371/journal.pone.0296285 (PMC10745165; doi:10.1371/journal.pone.0296285)
Supplement: S2 Table — Data are presented as n values; Abbreviations: MVPA = moderate-to-vigorous physical activity, LPA = light physical activity, SB = sedentary behavior. (DOCX) [file pone.0296285.s002.docx]

|  | **Sitting** | **Part-time** **Mixed** | **Active** | **Non-Working** |
| --- | --- | --- | --- | --- |
| **SB, Prolonged SB, and Steps per day** | | | | |
| 1^st^ Trimester | 59 | 8 | 27 | 28 |
| 2^nd^ Trimester | 57 | 9 | 27 | 25 |
| 3^rd^ Trimester | 56 | 8 | 24 | 23 |
| **LPA** | | | | |
| 1^st^ Trimester | 58 | 7 | 26 | 27 |
| 2^nd^ Trimester | 57 | 7 | 27 | 24 |
| 3^rd^ Trimester | 54 | 7 | 23 | 21 |
| **MVPA** | | | | |
| 1^st^ Trimester | 59 | 8 | 27 | 28 |
| 2^nd^ Trimester | 58 | 7 | 28 | 24 |
| 3^rd^ Trimester | 54 | 8 | 23 | 22 |

**Supplemental Table 2. Sample sizes for All-day Activity Data by Group as Presented in Table 2**

Data are presented as n values; Abbreviations: MVPA=moderate-to-vigorous physical activity, LPA=light physical activity, SB=sedentary behavior
