# Supplementary material for: Occupational physical activity as a determinant of daytime activity patterns and pregnancy and infant health
Source: PLoS One. 2023 Dec 22;18(12):e0296285. doi: 10.1371/journal.pone.0296285 (PMC10745165; doi:10.1371/journal.pone.0296285)
Supplement: S3 Table — Abbreviations: BIC = Bayesian Information Criteria, AIC = Akaike Information Criterion. (DOCX) [file pone.0296285.s003.docx]

**Supplemental Table 3. Statistics for Different Latent Class Models from One to Four Classes**

| Number of Classes | AIC | BIC | Class 1 N | Class 2 N | Class 3 N | Class 4 N | Model Interpretability |
| --- | --- | --- | --- | --- | --- | --- | --- |
| 1 | 2,030 | 2,045 | 99 | - | - | - | Not inclusive of individuals with different OPA patterns to answer research question |
| 2 | 1,967 | 1,993 | 70 | 29 | - | - | Not inclusive of individuals with different OPA patterns to answer research question |
| 3 | 1,957 | 1,993 | 61 | 9 | 29 | - | Generates classes that are inclusive of common OPA patterns and different hours worked a week |
| 4 | 1,896 | 1,943 | 57 | 9 | 26 | 7 | Generates two classes that have small sample sizes and class 2 and 3 are similar in the amount of time in OPA activities |

Abbreviations: BIC= Bayesian Information Criteria, AIC= Akaike Information Criterion
