# Supplementary material for: Occupational physical activity as a determinant of daytime activity patterns and pregnancy and infant health
Source: PLoS One. 2023 Dec 22;18(12):e0296285. doi: 10.1371/journal.pone.0296285 (PMC10745165; doi:10.1371/journal.pone.0296285)
Supplement: S4 Table — Data presented as n (%) or mean (SD); Abbreviations: BMI = body mass index, SD = standard deviation, PA = physical activity, MET = metabolic equivalents, p-values reflect a hypothesis test across study sites using one-way ANVOA for continuous variables or chi-square for categorical variables; Bold p-values indicate statistical significance. (DOCX) [file pone.0296285.s004.docx]

|  | **Overall**  (n=131) | **Pittsburgh Site**  (MoM Health: n=111) | **Iowa Site**  (PRAMS: n=20) | **p-value** |
| --- | --- | --- | --- | --- |
| **Age (years)** | 30.9 ± 4.9 | 30.9 (5.0) | 30.5 (4.3) | 0.687 |
| **Pre-Pregnancy BMI (kg/m^2^)** | 26.8 ± 6.7 | 26.4 (6.8) | 29.2 (5.6) | 0.086 |
| **Race** |  |  |  | 0.077 |
| **White** | 101 (77.1%) | 82 (73.9) | 19 (95.0) |  |
| **Black** | 23 (17.6%) | 23 (20.7) | 0 (0) |  |
| **Other** | 7 (5.3%) | 6 (5.4) | 1 (5.0) |  |
| **Education** |  |  |  | 0.510 |
| **Less than a Bachelor’s Degree** | 44  (33.6%) | 36 (32.4) | 8 (40.0) |  |
| **Bachelor’s Degree or Higher** | 87  (66.4%) | 75 (67.7) | 12 (60.0 |  |
| **Self-Reported Leisure-time PA (MET-h/week)** | | | | |
| **1^st^ Trimester (n=130)** | 17.0  (14.9) | 17.7 (15.3) | 13.0 (9.1) | 0.198 |
| **2^nd^ Trimester (n=121)** | 15.3  (13.9) | 17.2 (14.2) | 5.0 (4.3) | **<0.001** |
| **3^rd^ Trimester (n=117)** | 11.9  (13.4) | 13.3 (14.1) | 4.5 (4.6) | **0.008** |

**Supplemental Table 4. Participant Characteristics by Site**

Data presented as n (%) or mean (SD); Abbreviations: BMI=body mass index, SD= standard deviation, PA=physical activity, MET=metabolic equivalents, p-values reflect a hypothesis test across study sites using one-way ANVOA for continuous variables or chi-square for categorical variables; Bold p-values indicate statistical significance
