# Supplementary material for: Occupational physical activity as a determinant of daytime activity patterns and pregnancy and infant health
Source: PLoS One. 2023 Dec 22;18(12):e0296285. doi: 10.1371/journal.pone.0296285 (PMC10745165; doi:10.1371/journal.pone.0296285)
Supplement: S5 Table — Abbreviations: APO = any adverse pregnancy outcome, HDP = hypertensive disorders of pregnancy, GWG = gestational weight gain, IUGR = intrauterine growth restriction, GDM = gestational diabetes mellitus 95% CI = 95% confidence interval; **Likelihood ratio test evaluating all groups; Adjusted for age, pre-pregnancy BMI, race, and education level; ***Likelihood ratio test excluding the part-time mixed group due to no events;—indicates odds ratios and likelihood test were not conducted due to low number of events. (DOCX) [file pone.0296285.s005.docx]

**Supplemental Table 5. Frequency and Odds of Experiencing an APO by Group**

|  | **N by Group** | **Number of Events by Group**  (% of events within group) | **Odds Ratio**  (95% CI) | **p-value** |
| --- | --- | --- | --- | --- |
| **Any APO**  (n=30, 23.3%) | 31 | Non-working (n=5, 16.1%) | 1.0 (reference) | 0.416****** |
|  | 60 | Sitting (n=15, 25.0%) | 2.27 (0.63-8.18) |  |
|  | 9 | Part-time mixed (n=1, 11.1%) | 0.86 (0.08-9.1) |  |
|  | 29 | Active (n=9, 31.0%) | 2.40 (0.66-9.75) |  |
| **HDP**  (n=23, 18.1%) | 31 | Non-working (n=4, 12.9%) | 1.0 (reference) | 0.306*** |
|  | 58 | Sitting (n=11, 19.0%) | 2.08 (0.51-8.57) |  |
|  | 9 | Part-time mixed (n=0, 0.0%) | - |  |
|  | 29 | Active (n=8, 37.6%) | 2.88 (0.71-11.6) |  |
| **Excessive GWG**  (n=60, 48.4%) | 29 | Non-working (n=11, 37.9%) | 1.0 (reference) | 0.133****** |
|  | 59 | Sitting (n=32, 54.2%) | 1.95 (0.63-6.03) |  |
|  | 8 | Part-time mixed (n=1, 12.5%) | 0.24 (0.02-2.48) |  |
|  | 28 | Active (n=16, 57.1%) | 1.81 (0.53-6.19) |  |
| **Preterm Birth**  (n= 7, 5.6%) | 29 | Non-working (n=2, 6.9%) | - | - |
|  | 59 | Sitting (n=4, 6.8%) | - |  |
|  | 9 | Part-time mixed (n=0, 0.0%) | - |  |
|  | 29 | Active (n=1, 3.4%) | - |  |
| **IUGR**  (n= 4, 3.2%) | 31 | Non-working (n=1, 3.2%) | - | - |
|  | 56 | Sitting (n=3, 5.4%) | - |  |
|  | 9 | Part-time mixed (n=0, 0.0%) | - |  |
|  | 29 | Active (n=0, 0.0%) | - |  |
| **GDM**  (n= 6, 4.7%) | 31 | Non-working (n=0, 0.0%) | - | - |
|  | 60 | Sitting (n=2, 3.3%) | - |  |
|  | 8 | Part-time mixed (n=1, 12.5%) | - |  |
|  | 28 | Active (n=3, 10.7%) | - |  |

Abbreviations: APO= any adverse pregnancy outcome, HDP= hypertensive disorders of pregnancy, GWG= gestational weight gain, IUGR= intrauterine growth restriction, GDM= gestational diabetes mellitus 95% CI= 95% confidence interval; **Likelihood ratio test evaluating all groups; Adjusted for age, pre-pregnancy BMI, race, and education level; ***Likelihood ratio test excluding the part-time mixed group due to no events; - indicates odds ratios and likelihood test were not conducted due to low number of events
